# Dichlorido(2,9-dimethyl-4,7-diphenyl-1,10-phenanthroline-κ^2^ *N*,*N*′)mercury(II) acetonitrile hemisolvate

**DOI:** 10.1107/S1600536809024180

**Published:** 2009-07-01

**Authors:** Roya Ahmadi, Khadijeh Kalateh, Robabeh Alizadeh, Zeinab Khoshtarkib, Vahid Amani

**Affiliations:** a Islamic Azad University, Shahr-e-Rey Branch, Tehran, Iran; b School of Chemistry, Damghan University of Basic Sciences, Damghan, Iran

## Abstract

The asymmetric unit of the title compound, [HgCl_2_(C_26_H_20_N_2_)]·0.5CH_3_CN, contains two crystallographic­ally independent [HgCl_2_(C_26_H_20_N_2_)] mol­ecules and one acetonitrile solvent mol­ecule. The Hg^II^ atoms are four-coordin­ated in distorted tetra­hedral configurations by two N atoms from 2,9-dimethyl-4,7-diphenyl-1,10-phenanthroline ligands and two Cl atoms. The ligand ring systems are not planar. The phenyl rings are oriented at dihedral angles of 74.61 (3) and 66.00 (3)° in the two molecules. In the crystal structure, π–π contacts between phenanthroline rings [centroid–centroid distances = 3.809 (1), 3.686 (1), 3.986 (1), 3.877 (1), 3.697 (1), 3.789 (1), 3.745 (1), 3.797 (1) and 3.638 (1) Å] may stabilize the structure.

## Related literature

For Hg^II^ *X*_2_ complexes (*X*=Br, Cl, I and SCN) with bidentate *N*,*N*′ donor sets, see: Ahmadi *et al.* (2008 ▶); Alizadeh (2009 ▶); Hughes *et al.* (1985 ▶); Kalateh *et al.* (2008 ▶); Khoshtarkib *et al.* (2009 ▶); Mahjoub & Morsali (2003 ▶); Morsali (2006 ▶); Morsali *et al.* (2003 ▶, 2004 ▶); Safari *et al.* (2009 ▶); Tadayon Pour *et al.* (2008 ▶); Xie *et al.* (2004 ▶); Yousefi *et al.* (2009 ▶); Yousefi, Rashidi Vahid *et al.* (2008 ▶); Yousefi, Tadayon Pour *et al.* (2008 ▶). 
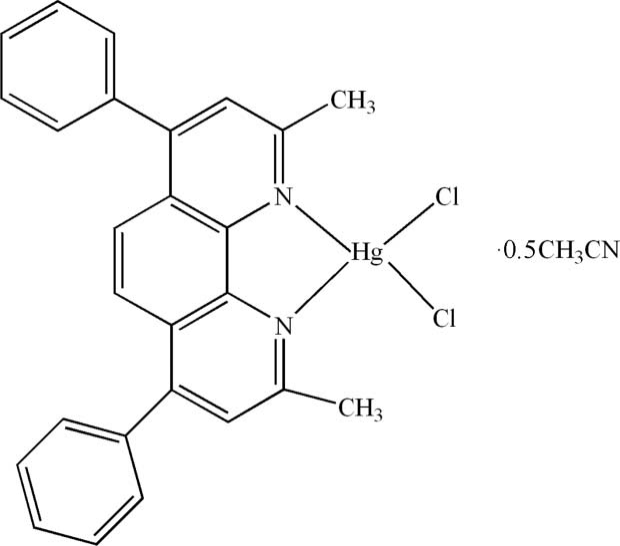


## Experimental

### 

#### Crystal data

[HgCl_2_(C_26_H_20_N_2_)]·0.5C_2_H_3_N
*M* *_r_* = 652.46
Triclinic, 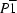

*a* = 11.7514 (6) Å
*b* = 14.1283 (7) Å
*c* = 16.1311 (8) Å
α = 107.537 (4)°
β = 101.924 (4)°
γ = 100.138 (4)°
*V* = 2415.8 (2) Å^3^
*Z* = 4
Mo *K*α radiation
μ = 6.61 mm^−1^
*T* = 120 K
0.50 × 0.13 × 0.11 mm

#### Data collection

Bruker SMART CCD area-detector diffractometer
Absorption correction: multi-scan (*SADABS*; Bruker, 1998 ▶) *T*_min_ = 0.379, *T*_max_ = 0.479
27210 measured reflections
12974 independent reflections
11253 reflections with *I* > 2σ(*I*)
*R*_int_ = 0.040

#### Refinement

*R*[*F* ^2^ > 2σ(*F* ^2^)] = 0.033
*wR*(*F* ^2^) = 0.074
*S* = 1.06
12974 reflections
586 parameters
H-atom parameters constrained
Δρ_max_ = 1.80 e Å^−3^
Δρ_min_ = −1.65 e Å^−3^

### 

Data collection: *SMART* (Bruker, 1998 ▶); cell refinement: *SAINT* (Bruker, 1998 ▶); data reduction: *SAINT*; program(s) used to solve structure: *SHELXTL* (Sheldrick, 2008 ▶); program(s) used to refine structure: *SHELXTL*; molecular graphics: *ORTEP-3 for Windows* (Farrugia, 1997 ▶); software used to prepare material for publication: *WinGX* (Farrugia, 1999 ▶).

## Supplementary Material

Crystal structure: contains datablocks I, global. DOI: 10.1107/S1600536809024180/hk2718sup1.cif

Structure factors: contains datablocks I. DOI: 10.1107/S1600536809024180/hk2718Isup2.hkl

Additional supplementary materials:  crystallographic information; 3D view; checkCIF report

## Figures and Tables

**Table 1:** Selected geometric parameters (Å, °)

Hg1—Cl2	2.3892 (10)
Hg1—Cl1	2.3921 (11)
Hg2—Cl4	2.3847 (12)
Hg2—Cl3	2.4420 (11)
N1—Hg1	2.337 (3)
N2—Hg1	2.331 (3)
N3—Hg2	2.328 (3)
N4—Hg2	2.319 (3)

**Fig. 1.**
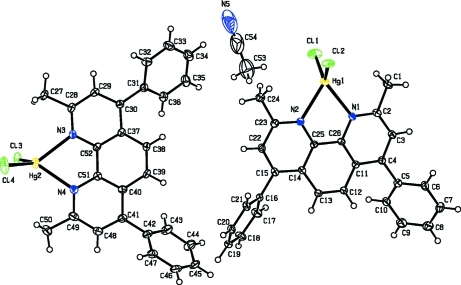
The molecular structure of the title molecule, with the atom-numbering scheme. Displacement ellipsoids are drawn at the 40% probability level.

**Fig. 2.**
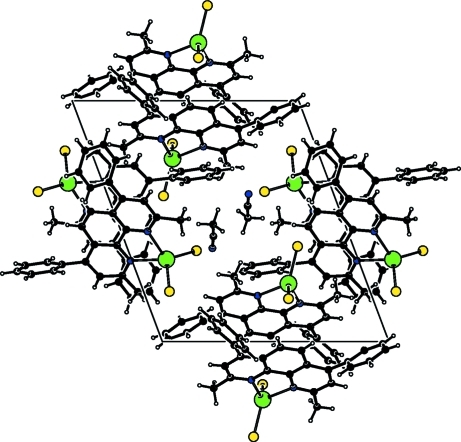
A partial packing diagram of the title compound.

## supplementary crystallographic information

### Comment

There are several Hg^II^ complexes, with formula, [Hg(N—N)*X*_2_], (*X*=Br, Cl, I and SCN), such as [Hg(2,9-dmphen)Br_2_], (II), (Khoshtarkib *et al.*, 2009), [Hg(ph_2_dmphen)SCN_2_], (III), (Alizadeh 2009), [Hg(TPA)Br_2_], (IV), (Xie *et al.*, 2004), [Hg(TPD)Br_2_], (V), (Hughes *et al.*, 1985), [Hg(NH(py)_2_)Br_2_], (VI), (Kalateh *et al.*, 2008), [Hg(6-mbpy)Cl_2_], (VII), (Ahmadi *et al.*, 2008), [Hg(NH(py)_2_)Cl_2_], (VIII), (Yousefi *et al.*, 2009), [Hg(4,4'-dmbpy)I_2_], (IX), (Yousefi, Tadayon Pour *et al.*, 2008), [Hg(5,5'-dmbpy)I_2_], (X), (Tadayon Pour *et al.*, 2008), [Hg(ph_2_phen)I_2_], (XI), (Yousefi, Rashidi Vahid *et al.*, 2008), [Hg(SCN)_2_(TBI)], (XII), (Morsali 2006), [Hg (dp4bt)(SCN)_2_], (XIII), (Mahjoub & Morsali 2003), [Hg(da4bt)(SCN)_2_], (XIV), (Morsali *et al.*, 2003), [Hg(biq)(SCN)_2_].C_6_H_6_, (XV), (Morsali *et al.*, 2004) and [Hg(dm4bt)(SCN)_2_], (XVI), (Safari *et al.*, 2009) [where 2,9-dmphen is 2,9-dimethyl-1,10-phenanthroline, ph_2_dmphen is 2,9-dimethyl-4,7-diphenyl-1,10-phenanthroline, TPA is tris(2-pyridyl)amine, TPD is *N*,*N*,*N*',*N*'-Tetramethyl-*o*-phenylenediamine, NH(py)_2_ is di-2-pyridylamine, 6-mbpy is 6-methyl-2,2'-bipyridine, 4,4'-dmbpy is 4,4'-dimethyl-2,2'-bipyridine, 5,5'-dmbpy is 5,5'-dimethyl-2,2'-bipyridine, ph_2_phen is 4,7-diphenyl-1,10-phenanthroline, TBI is 4,4',5,5'-tetramethyl-2,2'-bi-imidazole, dp4bt is 2,2'-diphenyl-4,4'-bithiazole, da4bt is 2,2'-diamino-4,4'-bithiazole, biq is 2,2'-biquinoline and dm4bt is 2,2'-dimethyl-4,4'-bithiazole] have been synthesized and characterized by single-crystal X-ray diffraction methods. We report herein the synthesis and crystal structure of the title compound, (I).

The asymmetric unit of the title compound, (I), (Fig. 1) contains two crystallographically independent [HgCl_2_(C_26_H_20_N_2_)] molecules and one acetonitrile solvent molecule. The Hg^II^ atoms are four-coordinated in distorted tetrahedral configurations by two N atoms from 2,9-dimethyl-4,7-diphenyl-1,10-phenanthroline and two Cl atoms (Table 1). Rings A (N1/C2-C4/C11/C26), B (C11-C14/C25/C26), C (N2/C14/C15/C22/C23/C25) and F (N3/C28-C30/C37/C52), G (C37-C40/C51/C52), H (N4/C40/C41/C48/C49/C51) are, of course, planar and the dihedral angles between them are A/B = 4.00 (3), A/C = 6.08 (3), B/C = 3.52 (3) and F/G = 5.77 (3), F/H = 5.55 (3), G/H = 3.53 (3) °. So, the phenanthroline ring systems are not planar. The phenyl rings D (C5-C10), E (C16-C21) and I (C31-C36), J (C42-C47) are oriented at dihedral angles of D/E = 74.61 (3) and I/J = 66.00 (3) °.

In the crystal structure (Fig. 2), the π–π contacts between the phenanthroline rings, Cg1—Cg2^i^, Cg2—Cg2^i^, Cg1—Cg3^i^, Cg2—Cg9^ii^, Cg6—Cg7^iii^, Cg6—Cg8^iii^, Cg7—Cg7^iii^, Cg11—Cg2^i^ and Cg12—Cg7^iii^, [symmetry codes: (i) 2 - x, 1 - y, -z, (ii) 2 - x, 1 - y, 1 - z, (iii) 2 - x, -y, 1 - z, where Cg1, Cg2, Cg3, Cg6, Cg7, Cg8, Cg9, Cg11 and Cg12 are centroids of the rings A (N1/C2-C4/C11/C26), B (C11-C14/C25/C26), C (N2/C14/C15/C22/C23/C25), F (N3/C28-C30/C37/C52), G (C37-C40/C51/C52), H (N4/C40/C41/C48/C49/C51), I (C31-C36), K (Hg1/N1/N2/C25/C26) and L (Hg2/N3/N4/C51/C52), respectively] may stabilize the structure, with centroid-centroid distances of 3.809 (1), 3.686 (1), 3.986 (1), 3.877 (1), 3.697 (1), 3.789 (1), 3.745 (1), 3.797 (1) and 3.638 (1) Å.

### Experimental

For the preparation of the title compound, (I), a solution of 2,9-dimethyl-4,7-diphenyl-1,10-phenanthroline (0.36 g, 1.10 mmol) in HCCl_3_ (20 ml) was added to a solution of HgCl_2_ (0.30 g, 1.10 mmol) in acetonitrile (20 ml) and the resulting pale yellow solution was stirred for 20 min at room temperature. Then, it was left to evaporate slowly at room temperature. After one week, colorless needle crystals of the title compound were isolated (yield; 0.47 g, 72.0%).

### Refinement

H atoms were positioned geometrically, with C-H = 0.93 and 0.96 Å for aromatic and methyl H, respectively, and constrained to ride on their parent atoms, with U_iso_(H) = 1.2U_eq_(C).

### Crystal data

**Table:**

[HgCl_2_(C_26_H_20_N_2_)]·0.5C_2_H_3_N	*Z* = 4
*M**_r_* = 652.46	*F*(000) = 1260
Triclinic, *P*1	*D*_x_ = 1.794 Mg m^−^^3^
Hall symbol: -P 1	Mo *K*α radiation, λ = 0.71073 Å
*a* = 11.7514 (6) Å	Cell parameters from 2354 reflections
*b* = 14.1283 (7) Å	θ = 1.7–29.2°
*c* = 16.1311 (8) Å	µ = 6.61 mm^−^^1^
α = 107.537 (4)°	*T* = 120 K
β = 101.924 (4)°	Needle, colorless
γ = 100.138 (4)°	0.50 × 0.13 × 0.11 mm
*V* = 2415.8 (2) Å^3^	

### Data collection

**Table:**

Bruker SMART CCD area-detector diffractometer	12974 independent reflections
Radiation source: fine-focus sealed tube	11253 reflections with *I* > 2σ(*I*)
graphite	*R*_int_ = 0.040
φ and ω scans	θ_max_ = 29.2°, θ_min_ = 1.7°
Absorption correction: multi-scan (*SADABS*; Bruker, 1998)	*h* = −16→15
*T*_min_ = 0.379, *T*_max_ = 0.479	*k* = −14→19
27210 measured reflections	*l* = −22→22

### Refinement

**Table:**

Refinement on *F*^2^	Primary atom site location: structure-invariant direct methods
Least-squares matrix: full	Secondary atom site location: difference Fourier map
*R*[*F*^2^ > 2σ(*F*^2^)] = 0.033	Hydrogen site location: inferred from neighbouring sites
*wR*(*F*^2^) = 0.074	H-atom parameters constrained
*S* = 1.06	*w* = 1/[σ^2^(*F*_o_^2^) + (0.0284*P*)^2^ + 6.1108*P*] where *P* = (*F*_o_^2^ + 2*F*_c_^2^)/3
12974 reflections	(Δ/σ)_max_ = 0.069
586 parameters	Δρ_max_ = 1.80 e Å^−^^3^
0 restraints	Δρ_min_ = −1.65 e Å^−^^3^

### Special details

**Table:**

Geometry. All e.s.d.'s (except the e.s.d. in the dihedral angle between two l.s. planes) are estimated using the full covariance matrix. The cell e.s.d.'s are taken into account individually in the estimation of e.s.d.'s in distances, angles and torsion angles; correlations between e.s.d.'s in cell parameters are only used when they are defined by crystal symmetry. An approximate (isotropic) treatment of cell e.s.d.'s is used for estimating e.s.d.'s involving l.s. planes.
Refinement. Refinement of *F*^2^ against ALL reflections. The weighted *R*-factor *wR* and goodness of fit *S* are based on *F*^2^, conventional *R*-factors *R* are based on *F*, with *F* set to zero for negative *F*^2^. The threshold expression of *F*^2^ > σ(*F*^2^) is used only for calculating *R*-factors(gt) *etc*. and is not relevant to the choice of reflections for refinement. *R*-factors based on *F*^2^ are statistically about twice as large as those based on *F*, and *R*- factors based on ALL data will be even larger.

### Fractional atomic coordinates and isotropic or equivalent isotropic displacement parameters (Å^2^)

**Table:**

	*x*	*y*	*z*	*U*_iso_*/*U*_eq_	
Hg1	0.275010 (12)	0.652623 (11)	0.240510 (10)	0.02166 (4)	
Hg2	−0.094858 (14)	−0.153897 (11)	0.658801 (11)	0.02624 (4)	
Cl1	0.30469 (10)	0.75429 (10)	0.39530 (8)	0.0404 (3)	
Cl2	0.43961 (8)	0.62792 (9)	0.17808 (7)	0.0327 (2)	
Cl3	−0.17092 (11)	−0.11436 (8)	0.79185 (8)	0.0325 (2)	
Cl4	−0.01545 (15)	−0.30065 (10)	0.62270 (10)	0.0496 (3)	
N1	0.0961 (3)	0.6705 (2)	0.1589 (2)	0.0177 (6)	
N2	0.1302 (3)	0.4968 (2)	0.1904 (2)	0.0182 (6)	
N3	0.0021 (3)	0.0072 (2)	0.6640 (2)	0.0199 (6)	
N4	−0.2087 (3)	−0.1175 (2)	0.5423 (2)	0.0223 (6)	
N5	0.4397 (9)	0.6246 (9)	0.6124 (10)	0.148 (5)	
C1	0.1860 (4)	0.8503 (3)	0.1935 (3)	0.0293 (9)	
H1A	0.2523	0.8372	0.1696	0.035*	
H1B	0.2092	0.8648	0.2576	0.035*	
H1C	0.1639	0.9082	0.1817	0.035*	
C2	0.0812 (3)	0.7580 (3)	0.1493 (3)	0.0208 (7)	
C3	−0.0296 (3)	0.7638 (3)	0.1013 (3)	0.0197 (7)	
H3	−0.0383	0.8261	0.0961	0.024*	
C4	−0.1262 (3)	0.6783 (3)	0.0617 (2)	0.0176 (6)	
C5	−0.2449 (3)	0.6878 (3)	0.0150 (2)	0.0188 (7)	
C6	−0.2503 (3)	0.7394 (3)	−0.0462 (3)	0.0230 (7)	
H6	−0.1796	0.7703	−0.0553	0.028*	
C7	−0.3607 (4)	0.7453 (3)	−0.0941 (3)	0.0289 (8)	
H7	−0.3638	0.7788	−0.1358	0.035*	
C8	−0.4654 (4)	0.7006 (3)	−0.0789 (3)	0.0337 (10)	
H8	−0.5395	0.7024	−0.1118	0.040*	
C9	−0.4606 (4)	0.6536 (3)	−0.0155 (3)	0.0319 (9)	
H9	−0.5314	0.6261	−0.0044	0.038*	
C10	−0.3521 (3)	0.6467 (3)	0.0319 (3)	0.0217 (7)	
H10	−0.3499	0.6151	0.0748	0.026*	
C11	−0.1092 (3)	0.5835 (3)	0.0690 (2)	0.0164 (6)	
C12	−0.1984 (3)	0.4878 (3)	0.0245 (2)	0.0184 (6)	
H12	−0.2705	0.4855	−0.0140	0.022*	
C13	−0.1803 (3)	0.4005 (3)	0.0371 (2)	0.0179 (6)	
H13	−0.2401	0.3396	0.0068	0.022*	
C14	−0.0712 (3)	0.3999 (3)	0.0961 (2)	0.0167 (6)	
C15	−0.0507 (3)	0.3129 (3)	0.1169 (2)	0.0181 (6)	
C16	−0.1438 (3)	0.2144 (3)	0.0843 (2)	0.0189 (7)	
C17	−0.2524 (4)	0.2115 (3)	0.1062 (3)	0.0297 (9)	
H17	−0.2687	0.2723	0.1373	0.036*	
C18	−0.3376 (4)	0.1181 (3)	0.0819 (3)	0.0310 (9)	
H18	−0.4090	0.1166	0.0985	0.037*	
C19	−0.3150 (4)	0.0284 (3)	0.0333 (3)	0.0273 (8)	
H19	−0.3719	−0.0337	0.0162	0.033*	
C20	−0.2078 (4)	0.0306 (3)	0.0099 (3)	0.0250 (8)	
H20	−0.1933	−0.0299	−0.0238	0.030*	
C21	−0.1224 (4)	0.1227 (3)	0.0367 (3)	0.0227 (7)	
H21	−0.0495	0.1233	0.0227	0.027*	
C22	0.0597 (3)	0.3222 (3)	0.1730 (3)	0.0206 (7)	
H22	0.0748	0.2658	0.1874	0.025*	
C23	0.1493 (3)	0.4144 (3)	0.2088 (3)	0.0207 (7)	
C24	0.2690 (4)	0.4243 (3)	0.2706 (3)	0.0296 (9)	
H24A	0.2807	0.4762	0.3283	0.035*	
H24B	0.3319	0.4432	0.2446	0.035*	
H24C	0.2708	0.3599	0.2786	0.035*	
C25	0.0226 (3)	0.4915 (3)	0.1352 (2)	0.0159 (6)	
C26	0.0040 (3)	0.5840 (3)	0.1205 (2)	0.0156 (6)	
C27	0.1631 (4)	0.0257 (3)	0.7933 (3)	0.0278 (8)	
H27A	0.1831	−0.0359	0.7622	0.033*	
H27B	0.1092	0.0105	0.8275	0.033*	
H27C	0.2352	0.0757	0.8334	0.033*	
C28	0.1038 (3)	0.0675 (3)	0.7259 (3)	0.0207 (7)	
C29	0.1490 (3)	0.1676 (3)	0.7296 (2)	0.0217 (7)	
H29	0.2187	0.2088	0.7744	0.026*	
C30	0.0927 (3)	0.2067 (3)	0.6684 (2)	0.0191 (7)	
C31	0.1353 (3)	0.3159 (3)	0.6800 (2)	0.0213 (7)	
C32	0.2585 (4)	0.3629 (3)	0.7060 (3)	0.0277 (8)	
H32	0.3137	0.3245	0.7140	0.033*	
C33	0.2994 (4)	0.4670 (3)	0.7202 (3)	0.0349 (10)	
H33	0.3815	0.4975	0.7374	0.042*	
C34	0.2177 (5)	0.5249 (3)	0.7088 (3)	0.0347 (10)	
H34	0.2451	0.5941	0.7177	0.042*	
C35	0.0944 (4)	0.4795 (3)	0.6839 (3)	0.0314 (9)	
H35	0.0395	0.5184	0.6764	0.038*	
C36	0.0540 (4)	0.3758 (3)	0.6703 (3)	0.0256 (8)	
H36	−0.0282	0.3459	0.6544	0.031*	
C37	−0.0097 (3)	0.1394 (3)	0.5972 (2)	0.0177 (6)	
C38	−0.0654 (3)	0.1650 (3)	0.5221 (2)	0.0196 (7)	
H38	−0.0310	0.2259	0.5157	0.024*	
C39	−0.1681 (3)	0.1012 (3)	0.4602 (2)	0.0201 (7)	
H39	−0.2031	0.1194	0.4119	0.024*	
C40	−0.2237 (3)	0.0064 (3)	0.4677 (2)	0.0198 (7)	
C41	−0.3364 (3)	−0.0594 (3)	0.4072 (3)	0.0234 (7)	
C42	−0.4089 (3)	−0.0290 (3)	0.3382 (3)	0.0259 (8)	
C43	−0.4422 (4)	0.0639 (4)	0.3615 (3)	0.0339 (10)	
H43	−0.4161	0.1087	0.4212	0.041*	
C44	−0.5136 (4)	0.0894 (4)	0.2963 (3)	0.0393 (11)	
H44	−0.5376	0.1501	0.3129	0.047*	
C45	−0.5494 (4)	0.0260 (4)	0.2071 (3)	0.0339 (10)	
H45	−0.5960	0.0444	0.1634	0.041*	
C46	−0.5156 (4)	−0.0659 (4)	0.1825 (3)	0.0323 (9)	
H46	−0.5394	−0.1088	0.1222	0.039*	
C47	−0.4465 (3)	−0.0941 (3)	0.2477 (3)	0.0273 (8)	
H47	−0.4253	−0.1561	0.2311	0.033*	
C48	−0.3771 (4)	−0.1520 (3)	0.4168 (3)	0.0283 (8)	
H48	−0.4494	−0.1964	0.3778	0.034*	
C49	−0.3121 (4)	−0.1806 (3)	0.4842 (3)	0.0277 (8)	
C50	−0.3575 (4)	−0.2804 (4)	0.4959 (3)	0.0371 (10)	
H50A	−0.3709	−0.2671	0.5546	0.045*	
H50B	−0.2990	−0.3198	0.4905	0.045*	
H50C	−0.4316	−0.3181	0.4502	0.045*	
C51	−0.1652 (3)	−0.0261 (3)	0.5351 (2)	0.0189 (7)	
C52	−0.0537 (3)	0.0409 (3)	0.6004 (2)	0.0184 (6)	
C53	0.2214 (10)	0.5822 (8)	0.5133 (7)	0.096 (3)	
H53C	0.2079	0.5363	0.4526	0.115*	
H53B	0.2049	0.6459	0.5119	0.115*	
H53A	0.1691	0.5519	0.5421	0.115*	
C54	0.3439 (10)	0.6006 (10)	0.5628 (10)	0.114 (4)	

### Atomic displacement parameters (Å^2^)

**Table:**

	*U*^11^	*U*^22^	*U*^33^	*U*^12^	*U*^13^	*U*^23^
Hg1	0.01569 (6)	0.01918 (7)	0.02492 (7)	0.00266 (5)	0.00219 (5)	0.00375 (5)
Hg2	0.03505 (8)	0.02347 (7)	0.02754 (8)	0.01105 (6)	0.01244 (6)	0.01467 (6)
Cl1	0.0311 (5)	0.0489 (7)	0.0276 (5)	0.0045 (5)	0.0090 (4)	−0.0031 (5)
Cl2	0.0180 (4)	0.0390 (5)	0.0308 (5)	0.0043 (4)	0.0049 (3)	0.0007 (4)
Cl3	0.0474 (6)	0.0313 (5)	0.0378 (5)	0.0249 (4)	0.0250 (5)	0.0218 (4)
Cl4	0.0869 (10)	0.0407 (6)	0.0555 (8)	0.0402 (7)	0.0473 (7)	0.0331 (6)
N1	0.0149 (12)	0.0151 (13)	0.0240 (15)	0.0037 (10)	0.0062 (11)	0.0078 (11)
N2	0.0163 (13)	0.0166 (13)	0.0203 (14)	0.0054 (11)	0.0030 (11)	0.0050 (11)
N3	0.0215 (14)	0.0210 (14)	0.0213 (15)	0.0070 (11)	0.0073 (12)	0.0111 (12)
N4	0.0219 (14)	0.0215 (15)	0.0241 (15)	0.0037 (12)	0.0072 (12)	0.0093 (12)
N5	0.092 (6)	0.157 (10)	0.279 (15)	0.039 (6)	0.091 (9)	0.164 (11)
C1	0.0221 (17)	0.0165 (17)	0.043 (2)	−0.0005 (14)	0.0027 (16)	0.0093 (16)
C2	0.0188 (15)	0.0178 (16)	0.0235 (17)	0.0024 (13)	0.0060 (13)	0.0052 (13)
C3	0.0197 (15)	0.0154 (15)	0.0249 (17)	0.0043 (12)	0.0079 (13)	0.0072 (13)
C4	0.0161 (14)	0.0188 (16)	0.0199 (16)	0.0049 (12)	0.0065 (12)	0.0083 (13)
C5	0.0180 (15)	0.0175 (15)	0.0225 (17)	0.0071 (12)	0.0085 (13)	0.0060 (13)
C6	0.0235 (17)	0.0196 (16)	0.0293 (19)	0.0069 (14)	0.0083 (15)	0.0118 (15)
C7	0.0288 (19)	0.0268 (19)	0.034 (2)	0.0139 (16)	0.0054 (16)	0.0136 (17)
C8	0.0212 (18)	0.027 (2)	0.049 (3)	0.0110 (15)	0.0010 (18)	0.0107 (19)
C9	0.0171 (17)	0.032 (2)	0.051 (3)	0.0076 (15)	0.0110 (17)	0.018 (2)
C10	0.0185 (16)	0.0196 (16)	0.0303 (19)	0.0079 (13)	0.0081 (14)	0.0106 (14)
C11	0.0161 (14)	0.0177 (15)	0.0164 (15)	0.0046 (12)	0.0058 (12)	0.0063 (12)
C12	0.0168 (14)	0.0162 (15)	0.0204 (16)	0.0032 (12)	0.0024 (12)	0.0064 (13)
C13	0.0162 (14)	0.0163 (15)	0.0191 (16)	0.0026 (12)	0.0029 (12)	0.0054 (12)
C14	0.0168 (14)	0.0148 (15)	0.0185 (15)	0.0026 (12)	0.0065 (12)	0.0056 (12)
C15	0.0190 (15)	0.0150 (15)	0.0228 (17)	0.0051 (12)	0.0096 (13)	0.0073 (13)
C16	0.0190 (15)	0.0151 (15)	0.0219 (16)	0.0031 (12)	0.0021 (13)	0.0084 (13)
C17	0.0238 (18)	0.0223 (18)	0.041 (2)	0.0054 (15)	0.0099 (17)	0.0075 (17)
C18	0.0182 (17)	0.026 (2)	0.045 (3)	−0.0001 (15)	0.0087 (16)	0.0106 (18)
C19	0.0258 (18)	0.0171 (16)	0.030 (2)	−0.0028 (14)	−0.0057 (15)	0.0093 (15)
C20	0.035 (2)	0.0154 (16)	0.0207 (17)	0.0038 (14)	0.0030 (15)	0.0053 (13)
C21	0.0280 (18)	0.0168 (16)	0.0250 (18)	0.0057 (14)	0.0082 (15)	0.0091 (14)
C22	0.0220 (16)	0.0166 (15)	0.0258 (18)	0.0077 (13)	0.0072 (14)	0.0091 (14)
C23	0.0183 (15)	0.0208 (16)	0.0249 (18)	0.0084 (13)	0.0058 (13)	0.0088 (14)
C24	0.0221 (17)	0.0248 (19)	0.038 (2)	0.0076 (15)	−0.0004 (16)	0.0106 (17)
C25	0.0145 (14)	0.0168 (15)	0.0158 (15)	0.0053 (12)	0.0038 (12)	0.0043 (12)
C26	0.0161 (14)	0.0136 (14)	0.0152 (14)	0.0032 (12)	0.0047 (12)	0.0026 (12)
C27	0.0296 (19)	0.034 (2)	0.0245 (19)	0.0118 (16)	0.0036 (15)	0.0172 (16)
C28	0.0215 (16)	0.0263 (18)	0.0205 (17)	0.0112 (14)	0.0078 (13)	0.0126 (14)
C29	0.0200 (16)	0.0281 (18)	0.0171 (16)	0.0079 (14)	0.0034 (13)	0.0083 (14)
C30	0.0196 (15)	0.0209 (16)	0.0175 (16)	0.0052 (13)	0.0062 (13)	0.0069 (13)
C31	0.0259 (17)	0.0203 (16)	0.0170 (16)	0.0064 (14)	0.0052 (13)	0.0059 (13)
C32	0.0245 (18)	0.0258 (19)	0.0266 (19)	0.0032 (15)	0.0040 (15)	0.0043 (16)
C33	0.036 (2)	0.025 (2)	0.032 (2)	−0.0025 (17)	0.0045 (18)	0.0037 (17)
C34	0.049 (3)	0.0201 (19)	0.030 (2)	0.0033 (17)	0.0117 (19)	0.0041 (16)
C35	0.046 (2)	0.0225 (19)	0.030 (2)	0.0138 (17)	0.0155 (19)	0.0087 (16)
C36	0.0290 (19)	0.0240 (18)	0.0257 (19)	0.0084 (15)	0.0090 (15)	0.0096 (15)
C37	0.0192 (15)	0.0211 (16)	0.0150 (15)	0.0079 (13)	0.0063 (12)	0.0066 (13)
C38	0.0230 (16)	0.0190 (16)	0.0199 (16)	0.0084 (13)	0.0064 (13)	0.0093 (13)
C39	0.0221 (16)	0.0259 (17)	0.0151 (15)	0.0097 (14)	0.0033 (13)	0.0105 (13)
C40	0.0189 (15)	0.0195 (16)	0.0180 (16)	0.0046 (13)	0.0046 (13)	0.0026 (13)
C41	0.0191 (16)	0.0274 (19)	0.0229 (18)	0.0055 (14)	0.0050 (14)	0.0084 (15)
C42	0.0171 (16)	0.034 (2)	0.0239 (18)	0.0058 (15)	0.0012 (14)	0.0104 (16)
C43	0.029 (2)	0.038 (2)	0.029 (2)	0.0138 (18)	−0.0002 (17)	0.0058 (18)
C44	0.031 (2)	0.046 (3)	0.040 (3)	0.018 (2)	0.0034 (19)	0.014 (2)
C45	0.0204 (17)	0.048 (3)	0.030 (2)	0.0050 (17)	−0.0049 (16)	0.020 (2)
C46	0.0191 (17)	0.045 (3)	0.0239 (19)	−0.0032 (17)	−0.0005 (15)	0.0105 (18)
C47	0.0198 (16)	0.033 (2)	0.0257 (19)	0.0022 (15)	0.0051 (14)	0.0090 (16)
C48	0.0242 (18)	0.0262 (19)	0.029 (2)	−0.0003 (15)	0.0034 (15)	0.0081 (16)
C49	0.0259 (18)	0.0241 (18)	0.029 (2)	−0.0012 (15)	0.0079 (15)	0.0072 (16)
C50	0.038 (2)	0.031 (2)	0.039 (2)	−0.0032 (18)	0.0068 (19)	0.0168 (19)
C51	0.0184 (15)	0.0200 (16)	0.0199 (16)	0.0063 (13)	0.0081 (13)	0.0067 (13)
C52	0.0173 (15)	0.0233 (17)	0.0168 (15)	0.0094 (13)	0.0048 (12)	0.0076 (13)
C53	0.132 (9)	0.093 (7)	0.089 (6)	0.029 (6)	0.055 (6)	0.050 (5)
C54	0.083 (6)	0.143 (10)	0.176 (12)	0.030 (7)	0.061 (8)	0.124 (10)

### Geometric parameters (Å, °)

**Table:**

Hg1—Cl2	2.3892 (10)	C26—N1	1.359 (4)
Hg1—Cl1	2.3921 (11)	C27—C28	1.496 (5)
Hg2—Cl4	2.3847 (12)	C27—H27A	0.9600
Hg2—Cl3	2.4420 (11)	C27—H27B	0.9600
N1—Hg1	2.337 (3)	C27—H27C	0.9600
N2—Hg1	2.331 (3)	C28—N3	1.336 (5)
N3—Hg2	2.328 (3)	C28—C29	1.399 (6)
N4—Hg2	2.319 (3)	C29—C30	1.380 (5)
C1—C2	1.496 (5)	C29—H29	0.9300
C1—H1A	0.9600	C30—C37	1.428 (5)
C1—H1B	0.9600	C30—C31	1.477 (5)
C1—H1C	0.9600	C31—C32	1.399 (5)
C2—N1	1.328 (5)	C31—C36	1.399 (6)
C2—C3	1.400 (5)	C32—C33	1.396 (6)
C3—C4	1.382 (5)	C32—H32	0.9300
C3—H3	0.9300	C33—C34	1.384 (7)
C4—C11	1.423 (5)	C33—H33	0.9300
C4—C5	1.491 (5)	C34—C35	1.396 (7)
C5—C6	1.391 (5)	C34—H34	0.9300
C5—C10	1.406 (5)	C35—C36	1.393 (6)
C6—C7	1.396 (5)	C35—H35	0.9300
C6—H6	0.9300	C36—H36	0.9300
C7—C8	1.386 (6)	C37—C52	1.416 (5)
C7—H7	0.9300	C37—C38	1.431 (5)
C8—C9	1.373 (7)	C38—C39	1.357 (5)
C8—H8	0.9300	C38—H38	0.9300
C9—C10	1.381 (5)	C39—C40	1.435 (5)
C9—H9	0.9300	C39—H39	0.9300
C10—H10	0.9300	C40—C51	1.403 (5)
C11—C26	1.414 (5)	C40—C41	1.434 (5)
C11—C12	1.430 (5)	C41—C48	1.378 (6)
C12—C13	1.354 (5)	C41—C42	1.474 (6)
C12—H12	0.9300	C42—C47	1.398 (6)
C13—C14	1.433 (5)	C42—C43	1.401 (6)
C13—H13	0.9300	C43—C44	1.382 (6)
C14—C15	1.415 (5)	C43—H43	0.9300
C14—C25	1.416 (4)	C44—C45	1.374 (7)
C15—C22	1.380 (5)	C44—H44	0.9300
C15—C16	1.482 (5)	C45—C46	1.392 (7)
C16—C21	1.388 (5)	C45—H45	0.9300
C16—C17	1.390 (5)	C46—C47	1.392 (6)
C17—C18	1.400 (5)	C46—H46	0.9300
C17—H17	0.9300	C47—H47	0.9300
C18—C19	1.376 (6)	C48—C49	1.399 (6)
C18—H18	0.9300	C48—H48	0.9300
C19—C20	1.386 (6)	C49—N4	1.339 (5)
C19—H19	0.9300	C49—C50	1.499 (6)
C20—C21	1.382 (5)	C50—H50A	0.9600
C20—H20	0.9300	C50—H50B	0.9600
C21—H21	0.9300	C50—H50C	0.9600
C22—C23	1.398 (5)	C51—N4	1.351 (5)
C22—H22	0.9300	C51—C52	1.454 (5)
C23—N2	1.328 (5)	C52—N3	1.355 (5)
C23—C24	1.503 (5)	C53—C54	1.430 (15)
C24—H24A	0.9600	C53—H53C	0.9600
C24—H24B	0.9600	C53—H53B	0.9600
C24—H24C	0.9600	C53—H53A	0.9600
C25—N2	1.364 (4)	C54—N5	1.163 (15)
C25—C26	1.442 (5)		
			
N2—Hg1—N1	71.59 (10)	N2—C25—C14	122.0 (3)
N2—Hg1—Cl2	109.36 (8)	N2—C25—C26	117.9 (3)
N1—Hg1—Cl2	122.71 (8)	C14—C25—C26	120.1 (3)
N2—Hg1—Cl1	113.98 (8)	N1—C26—C11	121.8 (3)
N1—Hg1—Cl1	106.46 (8)	N1—C26—C25	118.5 (3)
Cl2—Hg1—Cl1	122.09 (4)	C11—C26—C25	119.6 (3)
N4—Hg2—N3	72.04 (11)	C28—C27—H27A	109.5
N4—Hg2—Cl4	118.96 (9)	C28—C27—H27B	109.5
N3—Hg2—Cl4	123.84 (9)	H27A—C27—H27B	109.5
N4—Hg2—Cl3	111.86 (9)	C28—C27—H27C	109.5
N3—Hg2—Cl3	103.17 (8)	H27A—C27—H27C	109.5
Cl4—Hg2—Cl3	118.10 (4)	H27B—C27—H27C	109.5
C26—N1—Hg1	115.7 (2)	N3—C28—C29	120.8 (3)
C23—N2—C25	119.9 (3)	N3—C28—C27	117.4 (3)
C23—N2—Hg1	124.0 (2)	C29—C28—C27	121.7 (3)
C25—N2—Hg1	116.0 (2)	C30—C29—C28	121.6 (3)
C28—N3—C52	119.6 (3)	C30—C29—H29	119.2
C28—N3—Hg2	124.7 (3)	C28—C29—H29	119.2
C52—N3—Hg2	115.6 (2)	C29—C30—C37	117.7 (3)
C49—N4—C51	120.0 (4)	C29—C30—C31	120.1 (3)
C49—N4—Hg2	124.0 (3)	C37—C30—C31	122.1 (3)
C51—N4—Hg2	116.0 (2)	C32—C31—C36	118.6 (4)
C2—C1—H1A	109.5	C32—C31—C30	120.4 (4)
C2—C1—H1B	109.5	C36—C31—C30	121.0 (3)
H1A—C1—H1B	109.5	C33—C32—C31	120.7 (4)
C2—C1—H1C	109.5	C33—C32—H32	119.7
H1A—C1—H1C	109.5	C31—C32—H32	119.7
H1B—C1—H1C	109.5	C34—C33—C32	120.1 (4)
N1—C2—C3	121.0 (3)	C34—C33—H33	120.0
N1—C2—C1	117.6 (3)	C32—C33—H33	120.0
C3—C2—C1	121.4 (3)	C33—C34—C35	120.1 (4)
C4—C3—C2	120.9 (3)	C33—C34—H34	120.0
C4—C3—H3	119.5	C35—C34—H34	120.0
C2—C3—H3	119.5	C36—C35—C34	119.7 (4)
C3—C4—C11	118.2 (3)	C36—C35—H35	120.1
C3—C4—C5	119.8 (3)	C34—C35—H35	120.1
C11—C4—C5	122.0 (3)	C35—C36—C31	120.9 (4)
C6—C5—C10	118.8 (3)	C35—C36—H36	119.6
C6—C5—C4	119.5 (3)	C31—C36—H36	119.6
C10—C5—C4	121.7 (3)	C52—C37—C30	117.3 (3)
C5—C6—C7	120.8 (4)	C52—C37—C38	119.2 (3)
C5—C6—H6	119.6	C30—C37—C38	123.4 (3)
C7—C6—H6	119.6	C39—C38—C37	120.7 (3)
C8—C7—C6	119.2 (4)	C39—C38—H38	119.6
C8—C7—H7	120.4	C37—C38—H38	119.6
C6—C7—H7	120.4	C38—C39—C40	121.2 (3)
C9—C8—C7	120.4 (4)	C38—C39—H39	119.4
C9—C8—H8	119.8	C40—C39—H39	119.4
C7—C8—H8	119.8	C51—C40—C41	117.7 (3)
C8—C9—C10	120.9 (4)	C51—C40—C39	119.5 (3)
C8—C9—H9	119.6	C41—C40—C39	122.8 (3)
C10—C9—H9	119.6	C48—C41—C40	117.8 (4)
C9—C10—C5	119.8 (4)	C48—C41—C42	120.4 (4)
C9—C10—H10	120.1	C40—C41—C42	121.8 (4)
C5—C10—H10	120.1	C47—C42—C43	118.9 (4)
C26—C11—C4	117.7 (3)	C47—C42—C41	119.8 (4)
C26—C11—C12	118.5 (3)	C43—C42—C41	121.3 (4)
C4—C11—C12	123.7 (3)	C44—C43—C42	120.5 (4)
C13—C12—C11	121.6 (3)	C44—C43—H43	119.8
C13—C12—H12	119.2	C42—C43—H43	119.8
C11—C12—H12	119.2	C45—C44—C43	120.6 (5)
C12—C13—C14	121.5 (3)	C45—C44—H44	119.7
C12—C13—H13	119.3	C43—C44—H44	119.7
C14—C13—H13	119.3	C44—C45—C46	119.7 (4)
C15—C14—C25	117.8 (3)	C44—C45—H45	120.1
C15—C14—C13	124.0 (3)	C46—C45—H45	120.1
C25—C14—C13	118.2 (3)	C47—C46—C45	120.4 (4)
C22—C15—C14	118.1 (3)	C47—C46—H46	119.8
C22—C15—C16	118.9 (3)	C45—C46—H46	119.8
C14—C15—C16	123.0 (3)	C46—C47—C42	119.9 (4)
C21—C16—C17	118.5 (3)	C46—C47—H47	120.0
C21—C16—C15	121.4 (3)	C42—C47—H47	120.0
C17—C16—C15	120.0 (3)	C41—C48—C49	121.3 (4)
C16—C17—C18	120.8 (4)	C41—C48—H48	119.3
C16—C17—H17	119.6	C49—C48—H48	119.3
C18—C17—H17	119.6	N4—C49—C48	120.6 (4)
C19—C18—C17	119.5 (4)	N4—C49—C50	117.9 (4)
C19—C18—H18	120.2	C48—C49—C50	121.4 (4)
C17—C18—H18	120.2	C49—C50—H50A	109.5
C18—C19—C20	120.2 (4)	C49—C50—H50B	109.5
C18—C19—H19	119.9	H50A—C50—H50B	109.5
C20—C19—H19	119.9	C49—C50—H50C	109.5
C21—C20—C19	119.9 (4)	H50A—C50—H50C	109.5
C21—C20—H20	120.0	H50B—C50—H50C	109.5
C19—C20—H20	120.0	N4—C51—C40	122.5 (3)
C20—C21—C16	121.0 (4)	N4—C51—C52	118.3 (3)
C20—C21—H21	119.5	C40—C51—C52	119.2 (3)
C16—C21—H21	119.5	N3—C52—C37	122.6 (3)
C15—C22—C23	121.5 (3)	N3—C52—C51	118.1 (3)
C15—C22—H22	119.2	C37—C52—C51	119.3 (3)
C23—C22—H22	119.2	C54—C53—H53C	109.4
N2—C23—C22	120.8 (3)	C54—C53—H53B	109.5
N2—C23—C24	118.0 (3)	H53C—C53—H53B	109.5
C22—C23—C24	121.3 (3)	C54—C53—H53A	109.5
C23—C24—H24A	109.5	H53C—C53—H53A	109.5
C23—C24—H24B	109.5	H53B—C53—H53A	109.5
H24A—C24—H24B	109.5	N5—C54—C53	170.9 (16)
C23—C24—H24C	109.5	C2—N1—C26	120.3 (3)
H24A—C24—H24C	109.5	C2—N1—Hg1	124.0 (2)
H24B—C24—H24C	109.5		
			
N1—C2—C3—C4	−0.7 (6)	C39—C40—C41—C42	−6.1 (6)
C1—C2—C3—C4	−179.3 (4)	C48—C41—C42—C47	−54.9 (6)
C2—C3—C4—C11	−1.5 (5)	C40—C41—C42—C47	126.7 (4)
C2—C3—C4—C5	176.9 (3)	C48—C41—C42—C43	124.6 (5)
C3—C4—C5—C6	47.8 (5)	C40—C41—C42—C43	−53.8 (6)
C11—C4—C5—C6	−133.8 (4)	C47—C42—C43—C44	1.6 (7)
C3—C4—C5—C10	−131.5 (4)	C41—C42—C43—C44	−177.8 (4)
C11—C4—C5—C10	46.9 (5)	C42—C43—C44—C45	−2.4 (8)
C10—C5—C6—C7	−3.7 (6)	C43—C44—C45—C46	1.4 (8)
C4—C5—C6—C7	176.9 (4)	C44—C45—C46—C47	0.4 (7)
C5—C6—C7—C8	1.2 (6)	C45—C46—C47—C42	−1.1 (6)
C6—C7—C8—C9	1.9 (7)	C43—C42—C47—C46	0.1 (6)
C7—C8—C9—C10	−2.3 (7)	C41—C42—C47—C46	179.6 (4)
C8—C9—C10—C5	−0.3 (7)	C40—C41—C48—C49	1.0 (6)
C6—C5—C10—C9	3.3 (6)	C42—C41—C48—C49	−177.4 (4)
C4—C5—C10—C9	−177.4 (4)	C41—C48—C49—N4	1.0 (7)
C3—C4—C11—C26	3.1 (5)	C41—C48—C49—C50	178.8 (4)
C5—C4—C11—C26	−175.3 (3)	C41—C40—C51—N4	2.6 (5)
C3—C4—C11—C12	−174.3 (3)	C39—C40—C51—N4	−175.7 (3)
C5—C4—C11—C12	7.3 (6)	C41—C40—C51—C52	−177.0 (3)
C26—C11—C12—C13	5.9 (5)	C39—C40—C51—C52	4.7 (5)
C4—C11—C12—C13	−176.7 (4)	C30—C37—C52—N3	−4.5 (5)
C11—C12—C13—C14	0.4 (6)	C38—C37—C52—N3	172.9 (3)
C12—C13—C14—C15	175.3 (4)	C30—C37—C52—C51	173.3 (3)
C12—C13—C14—C25	−5.5 (5)	C38—C37—C52—C51	−9.3 (5)
C25—C14—C15—C22	−0.8 (5)	N4—C51—C52—N3	1.3 (5)
C13—C14—C15—C22	178.4 (3)	C40—C51—C52—N3	−179.0 (3)
C25—C14—C15—C16	176.6 (3)	N4—C51—C52—C37	−176.7 (3)
C13—C14—C15—C16	−4.2 (6)	C40—C51—C52—C37	3.0 (5)
C22—C15—C16—C21	−57.1 (5)	C3—C2—N1—C26	1.2 (6)
C14—C15—C16—C21	125.5 (4)	C1—C2—N1—C26	179.8 (4)
C22—C15—C16—C17	118.7 (4)	C3—C2—N1—Hg1	−178.3 (3)
C14—C15—C16—C17	−58.7 (5)	C1—C2—N1—Hg1	0.2 (5)
C21—C16—C17—C18	0.9 (6)	C11—C26—N1—C2	0.5 (5)
C15—C16—C17—C18	−175.0 (4)	C25—C26—N1—C2	−177.8 (3)
C16—C17—C18—C19	−2.1 (7)	C11—C26—N1—Hg1	−179.8 (3)
C17—C18—C19—C20	1.1 (7)	C25—C26—N1—Hg1	1.9 (4)
C18—C19—C20—C21	1.1 (6)	C22—C23—N2—C25	−0.7 (6)
C19—C20—C21—C16	−2.3 (6)	C24—C23—N2—C25	−179.4 (3)
C17—C16—C21—C20	1.3 (6)	C22—C23—N2—Hg1	−177.2 (3)
C15—C16—C21—C20	177.2 (4)	C24—C23—N2—Hg1	4.1 (5)
C14—C15—C22—C23	0.1 (6)	C14—C25—N2—C23	0.0 (5)
C16—C15—C22—C23	−177.4 (4)	C26—C25—N2—C23	177.3 (3)
C15—C22—C23—N2	0.7 (6)	C14—C25—N2—Hg1	176.7 (3)
C15—C22—C23—C24	179.4 (4)	C26—C25—N2—Hg1	−6.0 (4)
C15—C14—C25—N2	0.8 (5)	C29—C28—N3—C52	4.4 (5)
C13—C14—C25—N2	−178.4 (3)	C27—C28—N3—C52	−178.7 (3)
C15—C14—C25—C26	−176.4 (3)	C29—C28—N3—Hg2	−173.2 (3)
C13—C14—C25—C26	4.3 (5)	C27—C28—N3—Hg2	3.7 (5)
C4—C11—C26—N1	−2.7 (5)	C37—C52—N3—C28	−1.1 (5)
C12—C11—C26—N1	174.9 (3)	C51—C52—N3—C28	−179.0 (3)
C4—C11—C26—C25	175.5 (3)	C37—C52—N3—Hg2	176.7 (3)
C12—C11—C26—C25	−6.9 (5)	C51—C52—N3—Hg2	−1.2 (4)
N2—C25—C26—N1	2.8 (5)	C48—C49—N4—C51	−1.2 (6)
C14—C25—C26—N1	−179.8 (3)	C50—C49—N4—C51	−179.1 (4)
N2—C25—C26—C11	−175.5 (3)	C48—C49—N4—Hg2	178.5 (3)
C14—C25—C26—C11	1.8 (5)	C50—C49—N4—Hg2	0.7 (6)
N3—C28—C29—C30	−1.9 (6)	C40—C51—N4—C49	−0.6 (6)
C27—C28—C29—C30	−178.7 (4)	C52—C51—N4—C49	179.0 (4)
C28—C29—C30—C37	−3.8 (5)	C40—C51—N4—Hg2	179.6 (3)
C28—C29—C30—C31	173.5 (4)	C52—C51—N4—Hg2	−0.7 (4)
C29—C30—C31—C32	41.6 (5)	C23—N2—Hg1—N1	−178.4 (3)
C37—C30—C31—C32	−141.2 (4)	C25—N2—Hg1—N1	5.0 (2)
C29—C30—C31—C36	−134.9 (4)	C23—N2—Hg1—Cl2	62.5 (3)
C37—C30—C31—C36	42.3 (5)	C25—N2—Hg1—Cl2	−114.1 (2)
C36—C31—C32—C33	−1.2 (6)	C23—N2—Hg1—Cl1	−78.1 (3)
C30—C31—C32—C33	−177.7 (4)	C25—N2—Hg1—Cl1	105.3 (2)
C31—C32—C33—C34	0.2 (7)	C2—N1—Hg1—N2	176.1 (3)
C32—C33—C34—C35	0.6 (7)	C26—N1—Hg1—N2	−3.6 (2)
C33—C34—C35—C36	−0.3 (7)	C2—N1—Hg1—Cl2	−82.3 (3)
C34—C35—C36—C31	−0.8 (7)	C26—N1—Hg1—Cl2	98.1 (2)
C32—C31—C36—C35	1.6 (6)	C2—N1—Hg1—Cl1	65.7 (3)
C30—C31—C36—C35	178.1 (4)	C26—N1—Hg1—Cl1	−113.9 (2)
C29—C30—C37—C52	6.7 (5)	C49—N4—Hg2—N3	−179.7 (3)
C31—C30—C37—C52	−170.5 (3)	C51—N4—Hg2—N3	0.1 (3)
C29—C30—C37—C38	−170.5 (3)	C49—N4—Hg2—Cl4	61.0 (3)
C31—C30—C37—C38	12.2 (6)	C51—N4—Hg2—Cl4	−119.2 (3)
C52—C37—C38—C39	8.0 (5)	C49—N4—Hg2—Cl3	−82.3 (3)
C30—C37—C38—C39	−174.8 (4)	C51—N4—Hg2—Cl3	97.5 (3)
C37—C38—C39—C40	−0.2 (6)	C28—N3—Hg2—N4	178.3 (3)
C38—C39—C40—C51	−6.2 (6)	C52—N3—Hg2—N4	0.6 (2)
C38—C39—C40—C41	175.6 (4)	C28—N3—Hg2—Cl4	−68.4 (3)
C51—C40—C41—C48	−2.7 (5)	C52—N3—Hg2—Cl4	113.9 (2)
C39—C40—C41—C48	175.5 (4)	C28—N3—Hg2—Cl3	69.3 (3)
C51—C40—C41—C42	175.7 (4)	C52—N3—Hg2—Cl3	−108.5 (2)
